# An *NRAS* mutation in primary malignant melanoma of the lung: a case report

**DOI:** 10.1186/s13000-020-0928-8

**Published:** 2020-02-07

**Authors:** Takashi Hibiya, Meiro Tanaka, Mai Matsumura, Ayako Aoki, Tadashi Ikegami, Koji Okudela, Naomi Kawano, Kenichi Ohashi

**Affiliations:** 1 Department of Pathology, Yokohama City University Hospital, 3-9 Fukuura, Kanazawa-ku, Yokohama, 236-0004 Japan; 2 Department of Pathology, Yokohama Minami Kyousai Hospital, 1-21-1 Mutsuura-higashi, Kanazawa-ku, Yokohama, 236-0037 Japan; 3 Department of Pathology, Graduate School of Medicine, Yokohama City University, 3-9 Fukuura, Kanazawa-ku, Yokohama, 236-0004 Japan; 4 Department of Respiratory Medicine, Yokohama Minami Kyousai Hospital, 1-21-1 Mutsuura-higashi, Kanazawa-ku, Yokohama, 236-0037 Japan; 5 Department of Diagnostic Radiology, Kanagawa Dental University Hospital, 1-23 Ogawacho, Yokosuka, 238-8570 Japan; 6 Department of Radiology, Yokohama Minami Kyousai Hospital, 1-21-1 Mutsuura-higashi, Kanazawa-ku, Yokohama, 236-0037 Japan

**Keywords:** Primary malignant melanoma of the lung, *NRAS* mutation, Sanger sequencing, Autopsy

## Abstract

**Background:**

Primary malignant melanoma of the lung (PML) is extremely rare. No precursor lesions of PML have been identified, and little is known about the genetic mutations associated with the disease. Typically, 15–20% of malignant melanomas possess *NRAS* gene mutations, but no cases of *NRAS*-mutated PML have been reported in the English literature. We present a case of PML involving an *NRAS* mutation.

**Case presentation:**

Clinical summary

A 74-year-old Japanese female presented with worsening dyspnea and was admitted to hospital. Computed tomography (CT) revealed a right lung (S10) mass and pleural dissemination. Cytology of the pleural effusion in the right lung was performed, and malignant melanoma or clear cell sarcoma was suspected. A dermatological examination and gallium scintigraphy were conducted to determine the primary tumor site, but no suspicious lesions, expect for the right lung mass, were found. After admission, CT showed complicating bilateral pneumonia, and an antibiotic drug was administered, but the pleural effusion got worse. About 2 weeks later, the patient died of respiratory failure and cardiac arrest. An autopsy was performed to determine the histological diagnosis.

Autopsy findings

A 26x15x20-mm black and pale yellow mass was found in the right lower lobe. Many disseminated nodules were found in the right lobe. The tumor had invaded the right diaphragm. Subcarinal lymph node metastasis was also detected. Immunohistochemically, the tumor cells exhibited positivity for S-100 and HMB45 staining. The patient was diagnosed with malignant melanoma. Sanger sequencing of the tumor detected an *NRAS* mutation.

**Conclusions:**

We found an *NRAS* D54N mutation in PML, which has not been reported previously anywhere in the world. Previous reports indicated that most cases of PML can be classified into the triple-wild-type, but *BRAF* mutation status was only analyzed in a few cases. We should analyze the mutation patterns of PML to determine whether any subtypes other than the triple-wild-type exist. PML might be a form of de novo cancer.

## Background

Primary malignant melanoma of the lung is extremely rare and only accounts for 0.01% of all primary lung tumors [1]. The median age of patients with the condition is between 51 and 59 years, and the disease exhibits an approximately equal sex distribution or a slight male predominance [2]. No precursor lesions of primary malignant melanoma of the lung have been identified [2], and the associated genetic mutations are poorly understood. In one case, it was reported that pulmonary malignant melanoma carried a tumor protein p53 (*TP53*) mutation [3]. Typically, 15–20% of malignant melanomas have mutations in the neuroblastoma RAS viral oncogene homolog (*NRAS*) gene [4], but no cases of *NRAS*-mutated primary malignant melanoma of the lung have been reported in the English literature. We present the case of a 74-year-old female, who died of primary malignant melanoma of the lung involving an *NRAS* mutation.

## Case presentation

### Clinical summary

**Fig. 1:**
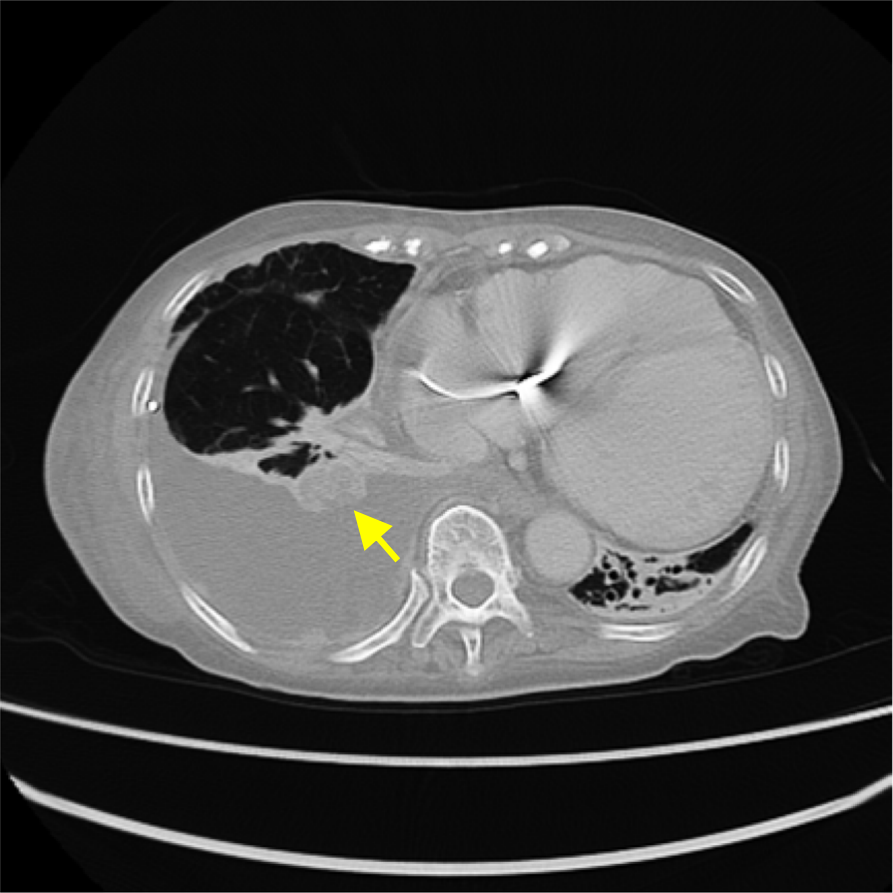
Chest CT indicated the presence of a right lung (S10) mass (arrow) and pleural dissemination

**Fig. 2:**
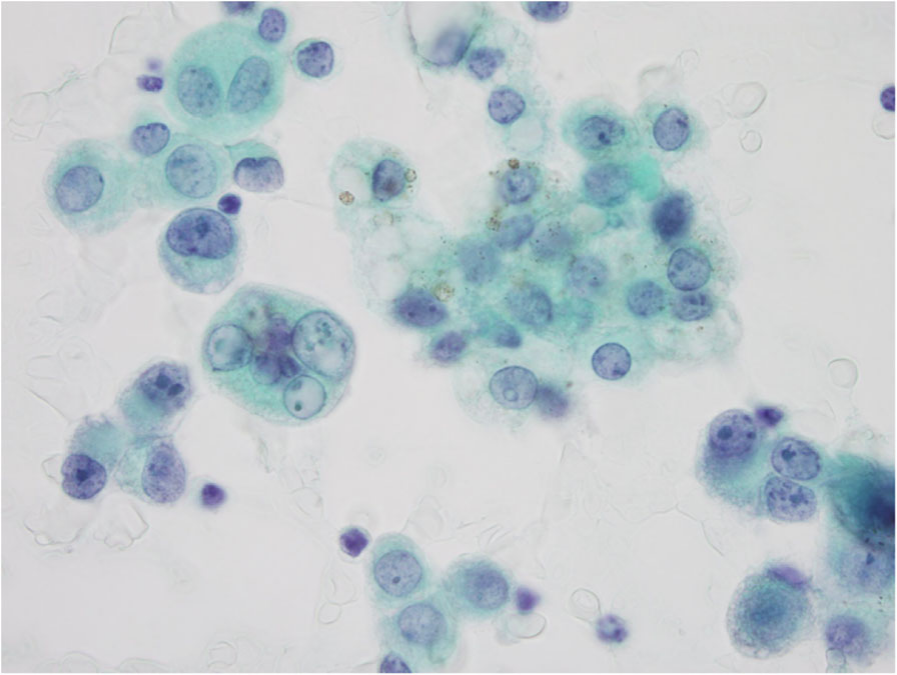
Cytology of the right pleural effusion showed malignant cells, which led us to suspect malignant melanoma or clear cell sarcoma

**Fig. 3:**
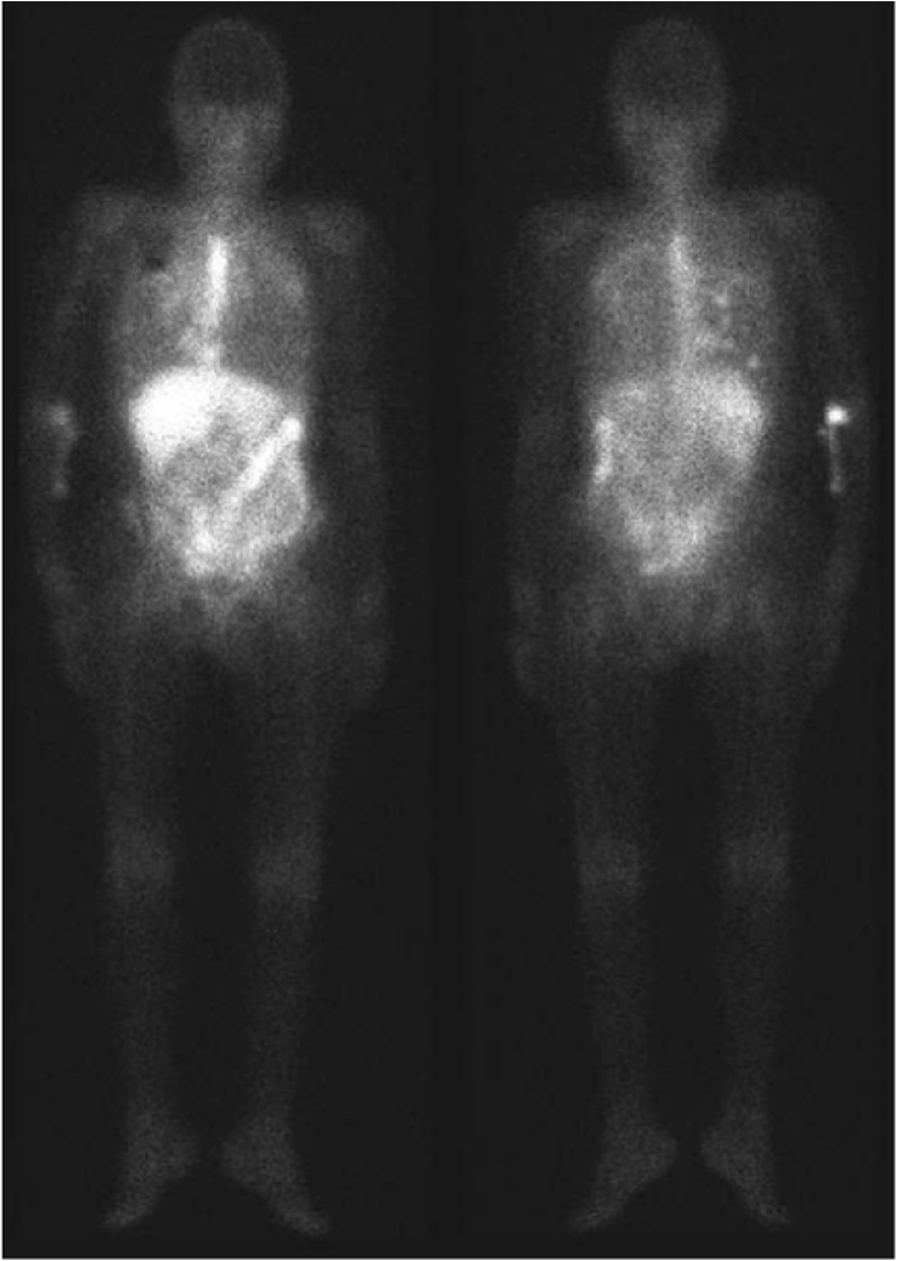
Gallium scintigraphy did not reveal any suspicious lesions expect the right lung mass

A 74-year-old Japanese female presented with worsening dyspnea and was admitted to hospital. A chest X-ray revealed right-sided pleural effusion and cardiac enlargement. Computed tomography (CT) showed a right lung (S10) mass and pleural dissemination (Fig. 1). Cytology of the pleural effusion in the right lung was performed, and tumor cells were obtained. The tumor cells had round nuclei, large and distinct nucleoli, and melanin particles in their cytoplasm. We suspected malignant melanoma or clear cell sarcoma (Fig. 2). A dermatological examination and gallium scintigraphy were conduced to determine the primary tumor site, but no suspicious lesions, expect the right lung mass, were found (Fig. 3).

After admission, CT showed ground-glass opacities in both lungs, and the patient was diagnosed with complicating bilateral pneumonia and was given an antibiotic drug. It was transiently effective, but the right pleural effusion got worse. About 2 weeks later, the patient died of respiratory failure and cardiac arrest. An autopsy was performed to determine the histological diagnosis.

### Autopsy findings

**Fig. 4:**
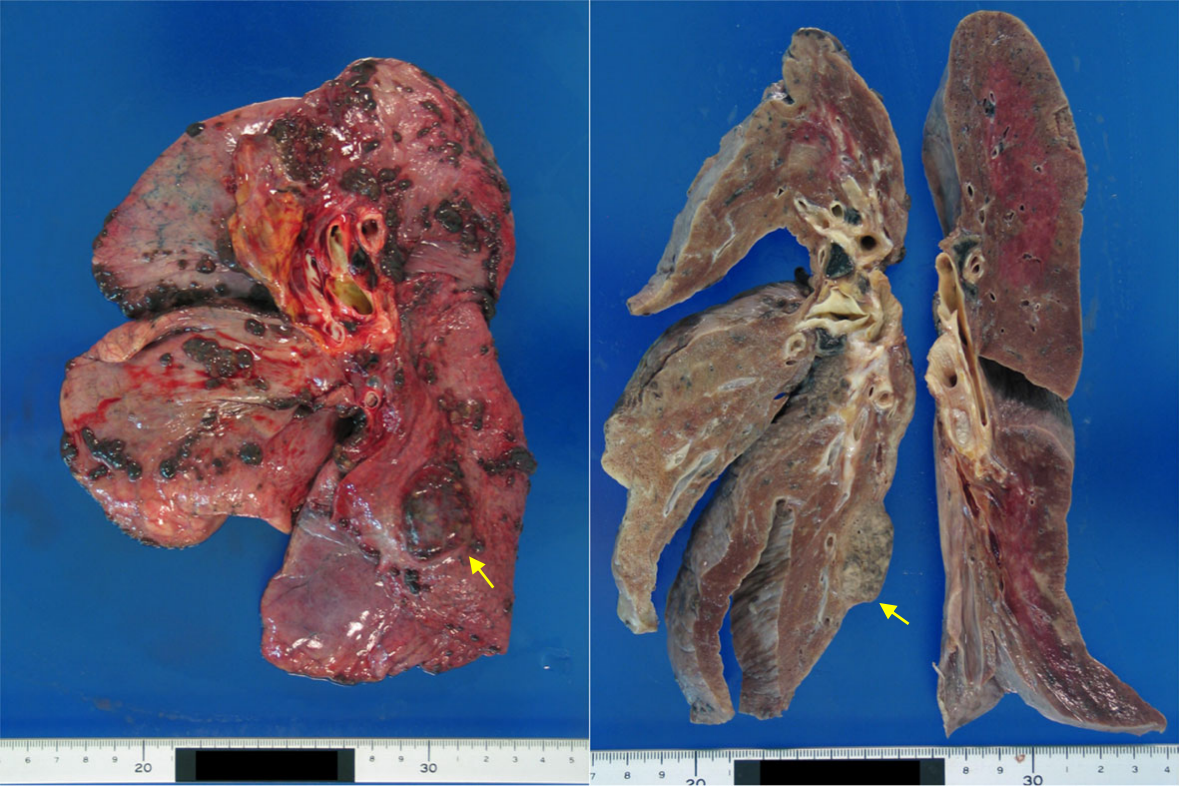
A 26x15x20-mm black and pale yellow mass was found in the right lower lobe. Many disseminated nodules were found in the right lobe

A 26x15x20-mm black and pale yellow mass was found in the right lower lobe. Many disseminated nodules were found in the right lobe (Fig. 4). The tumor had invaded the right diaphragm. A subcarinal lymph node metastasis (45x21x15 mm in size) was also detected. Pale blood-colored, massive right-sided pleural effusion (1850 ml) was noted, which was indicative of pleuritis carcinomatosa.

**Fig. 5:**
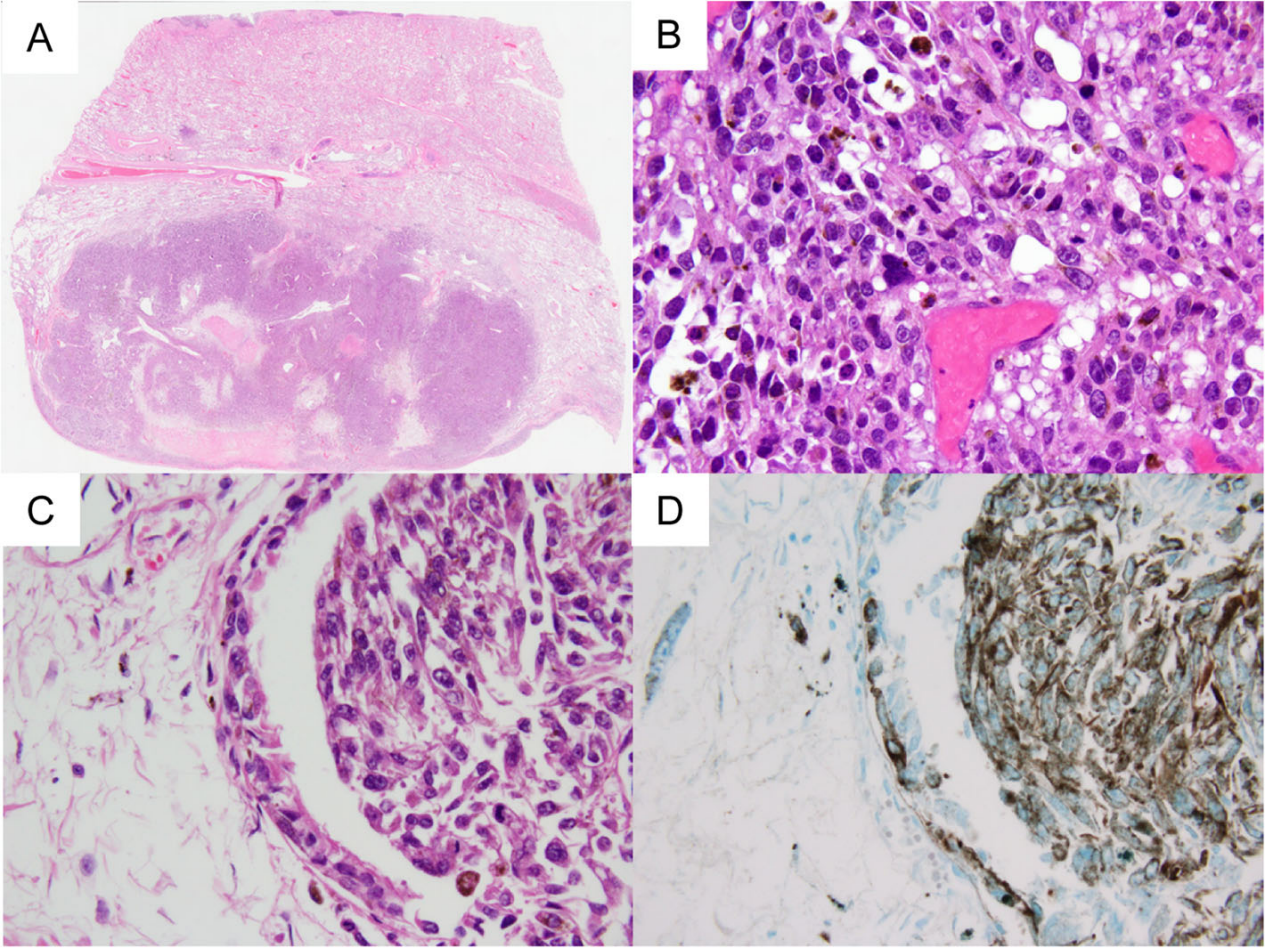
**a** and **b**: Malignant melanoma had invaded the right lower lobe. (Hematoxylin and eosin [HE] staining). **c** and **d**: The melanoma exhibited intraepithelial spread into a bronchus. (**c**: HE staining, **d**: HMB45 staining).

Histologically, the tumor cells had large nuclei with high nuclear/cytoplasmic ratios, large and distinct nucleoli, and melanin particles in their cytoplasm. The tumor exhibited intraepithelial spread into a bronchus (Fig. 5). No primary tumor was found, expect in the right lower lobe. We performed immunohistochemical staining using an HMB45 antibody and antibodies against S-100 and c-kit. The tumor cells exhibited positivity for S-100 and HMB45 staining (Fig. 5), but were negative for c-kit. The patient was diagnosed with malignant melanoma.

**Fig. 6:**
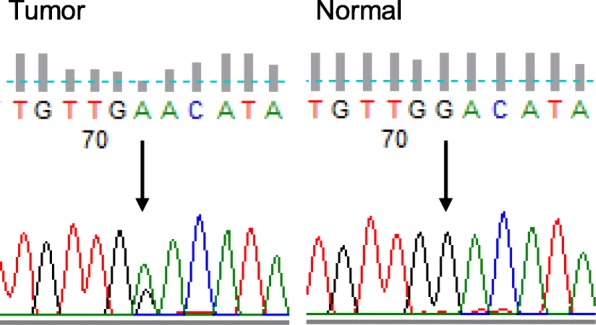
The results of *NRAS* exon 3 Sanger sequencing of the tumor and normal tissue are shown. The tumor carried a mutation

We determined the tumor’s proto-oncogene B-Raf (*BRAF*) and *NRAS* mutational status using Sanger sequencing. Primers were designed to amplify *BRAF* exon 15, *NRAS* exon 2, and *NRAS* exon 3 (Table 1). As a result, we detected an *NRAS* mutation (D54N) (Fig. 6). We also determined the *KIT* mutational status using Sanger sequencing. We sequenced *KIT* exon 8, 9, 11, 13, 17 and 18, but no *KIT* gene mutation was detected.

**Table 1:** Primers used for the Sanger sequencing

Gene	Exons	5′ → 3′ Sequence	Tm	Length
*BRAF*	EX15	F: TCATAATGCTTGCTCTGATAGG	60	224
		R: GGCCAAAAATTTAATCAGTGG		
*NRAS*	EX2	F: GAAAGCTTTAAAGTACTGTAGATGTGG	60	247
		R: AGATGATCCGACAAGTGAGAGA		
*NRAS*	EX3	F: CCCCTTACCCTCCACACC	60	243
		R: CACAAAGATCATCCTTTCAGAGAA

Apart from malignant melanoma, foamy macrophages exuded into the alveolar spaces of the bilateral lobes, and sputum had plugged the bronchi. Neutrophils had infiltrated into some alveoli and bronchi. We diagnosed the patient with lipoid pneumonia.

## Discussion

Primary malignant melanoma of the lung is extremely rare, and little is known about the genetic mutations associated with the condition. A previous study showed that malignant melanoma exhibited a high prevalence of somatic mutations [5]. However, somatic mutation patterns vary substantially between melanoma subtypes [6]. Sun-exposed skin melanomas have the highest numbers of mutations. Many have neurofibromatosis type I (*NF1*) and *NRAS* mutations, and sporadic *BRAF* V600K mutations are also seen [4, 7]. Moderately sun-exposed skin melanomas (excluding chronic sun-damaged skin melanomas) have intermediate numbers of mutations. Some possess the *BRAF* V600E mutation, whereas *NRAS* mutations are rare [4, 8]. Non-cutaneous melanomas have significantly lower numbers of mutations [4]. Ultraviolet (UV) light is cited as a possible major driver of mutagenesis in melanoma. As primary malignant melanoma of the lung is considered to be a non-UV related melanoma, it might exhibit few somatic mutations.

Previously, 73 cases of primary malignant melanoma of the lung have been reported in the English literature. However, mutation status was only analyzed in 9 cases (Table 2) [3, 9–15]. Of these 9 cases, all 9 were analyzed for *BRAF* mutations, but only 3 cases were analyzed for *NRAS* and *KIT* mutations, and only 2 cases were analyzed for *TP53* mutations. A *TP53* P72R mutation was detected in one case [3], but no *BRAF* or *NRAS* mutations were found. We detected an *NRAS* D54N mutation in our case, which is the first time an *NRAS* mutation has been detected in primary malignant melanoma of the lung anywhere in the world.

**Table 2:** Nine cases of primary malignant melanoma of the lung in which mutation status was analyzed

					Mutation			
No.	Author	Year	Age	Sex	*BRAF*	*NRAS*	*KIT*	*TP53*
1	dos Santos et al.	2013	62	F	Negative	N/A	N/A	N/A
2	Watanabe et al.	2015	66	M	Negative	Negative	Negative	P72R
3			46	F	Negative	Negative	Negative	Negative
4	Hirai et al.	2017	86	F	Negative	N/A	N/A	N/A
5	Kyriakopoulos et al.	2017	56	F	Negative	Negative	Negative	N/A
6	Yamamoto et al.	2017	61	F	Negative	N/A	N/A	N/A
7	Holmes and Chung	2017	43	F	Negative	N/A	N/A	N/A
8	Shi et al.	2018	46	M	Negative	N/A	N/A	N/A
9	Yabuki et al.	2018	74	M	Negative	N/A	N/A	N/A
10	Our case		74	F	Negative	D54N	Negative	N/A

Abbreviations: N/A indicates not available

Information about the genomic classification of malignant melanoma was previously reported in a classification of cutaneous melanoma [16]. According to the report, cutaneous melanoma can be classified into four subtypes: mutant *BRAF*; mutant *NRAS*; mutant *NF1*; and the triple-wild-type, which is characterized by a lack of hot-spot *BRAF*, *N/H/KRAS*, or *NF1* mutations. This genomic classification might aid the selection of therapeutic targets [4]. Almost all primary malignant melanomas of the lung are classified into the triple-wild-type, and few are classified into the mutant *NRAS* type. However, the mutation status of primary malignant melanoma of the lung has not been sufficiently analyzed yet. So, when we encounter malignant melanoma of the lung, we should analyze not only *BRAF* mutations, but also other mutations.

In our case, we detected an *NRAS* D54N mutation. Most of the *RAS* gene mutations found in cancer are missense mutations, with 98% of these mutations being located at the G12, G13, or Q61 hotspot [17]. D54N is an extremely rare mutation. In the Catalogue Of Somatic Mutations In Cancer (COSMIC) v88, the *NRAS* D54N mutation was only reported in one case of colon adenocarcinoma. The function of D54N-mutated *NRAS* is not yet known.

No precursor lesions of primary malignant melanoma of the lung have yet been identified [2]. Some of the authors previously reported a case of pulmonary melanocytic nevus. The nevus cells of the pulmonary melanocytic nevus exhibited a *BRAF* V600E mutation, but no *NRAS* mutations were found [18]. No *BRAF* mutations were detected in the 9 previously reported cases of primary malignant melanoma of the lung or our case; therefore, primary malignant melanoma of the lung might be a form of de novo cancer, rather than arise from melanocytic nevus.

## Conclusions

We found an *NRAS* D54N mutation in primary malignant melanoma of the lung, which has not been reported previously anywhere in the world. Previously reported cases have demonstrated that non-UV related melanomas have lower numbers of mutations than cutaneous melanoma, and most cases of primary malignant melanoma of the lung can be classified into the triple-wild-type because they are non-UV related tumors. However, the *BRAF* mutation status of primary malignant melanoma of the lung has only been analyzed in 9 previous cases, and other types of mutations have only been analyzed in 3 previous cases. We should analyze mutation patterns to determine whether some primary malignant melanomas of the lung belong to mutation subtypes other than the triple-wild-type.

Primary malignant melanoma of the lung might be a form of de novo cancer.

## Data Availability

Not applicable

## Abbreviations

COSMIC: Catalogue Of Somatic Mutations In Cancer
CT: Computed tomography
HE: Hematoxylin and eosin
*NF1*: Neurofibromatosis type I
*NRAS*: Neuroblastoma RAS viral oncogene homolog
PML: Primary malignant melanoma of the lung
*TP53*: Tumor protein p53
UV: Ultraviolet
